# Supplementary material for: Hot spot prediction in protein-protein interactions by an ensemble system
Source: BMC Syst Biol. 2018 Dec 31;12(Suppl 9):132. doi: 10.1186/s12918-018-0665-8 (PMC6311905; doi:10.1186/s12918-018-0665-8)
Supplement: Supplementary file 2 — All relevant MATLAB code. A simple Matlab implement of our predictor. (ZIP 19,742 kb) [file 12918_2018_665_MOESM2_ESM.zip › Additional file 2/Read ASA/ASA website/dbMPIKT/4EKCB.html]

 NetSurfP - Protein Surface Accessibility and Secondary Structure Predictions

  

|  |  |  |
| --- | --- | --- |
|  |  | NetSurfP - Protein Surface Accessibility and Secondary Structure Predictions  Technical University of Denmark |

  


---

```
# For publication of results, please cite:
# A generic method for assignment of reliability scores applied to solvent accessibility predictions.
# Bent Petersen, Thomas Nordahl Petersen, Pernille Andersen, Morten Nielsen and Claus Lundegaard
# BMC Structural Biology 2009, 9:51 doi:10.1186/1472-6807-9-51
#
# Column 1: Class assignment - B for buried or E for Exposed - Threshold: 25% exposure, but not based on RSA
# Column 2: Amino acid
# Column 3: Sequence name
# Column 4: Amino acid number
# Column 5: Relative Surface Accessibility - RSA
# Column 6: Absolute Surface Accessibility
# Column 7: Z-fit score for RSA prediction
# Column 8: Probability for Alpha-Helix
# Column 9: Probability for Beta-strand
# Column 10: Probability for Coil
E G  4EKCB                  1    0.927  72.947  -0.624   0.003   0.003   0.994
E E  4EKCB                  2    0.777 135.829  -1.943   0.018   0.019   0.964
E F  4EKCB                  3    0.617 123.832  -1.499   0.018   0.019   0.964
E G  4EKCB                  4    0.540  42.482  -2.021   0.018   0.019   0.964
E S  4EKCB                  5    0.582  68.152   0.385   0.016   0.005   0.979
B P  4EKCB                  6    0.269  38.143  -1.493   0.016   0.005   0.979
E S  4EKCB                  7    0.407  47.736   0.059   0.018   0.019   0.964
E P  4EKCB                  8    0.405  57.469  -0.150   0.858   0.002   0.139
E E  4EKCB                  9    0.647 113.118   0.495   0.923   0.002   0.076
E E  4EKCB                 10    0.345  60.271   0.645   0.923   0.002   0.076
B A  4EKCB                 11    0.061   6.700  -0.377   0.923   0.002   0.076
E Q  4EKCB                 12    0.437  77.977   0.083   0.858   0.002   0.139
E L  4EKCB                 13    0.510  93.399   0.967   0.782   0.003   0.216
B W  4EKCB                 14    0.111  26.720   0.077   0.694   0.003   0.303
B S  4EKCB                 15    0.214  25.081  -0.896   0.600   0.003   0.397
E E  4EKCB                 16    0.454  79.314   0.218   0.600   0.003   0.397
E A  4EKCB                 17    0.243  26.779   1.198   0.406   0.004   0.590
B F  4EKCB                 18    0.042   8.510   0.127   0.923   0.002   0.076
E D  4EKCB                 19    0.468  67.482   1.637   0.970   0.001   0.030
E E  4EKCB                 20    0.459  80.117   1.897   0.923   0.002   0.076
B L  4EKCB                 21    0.053   9.631   0.855   0.879   0.010   0.111
B L  4EKCB                 22    0.088  16.149   0.009   0.694   0.003   0.303
E A  4EKCB                 23    0.623  68.644   0.212   0.176   0.004   0.820
B S  4EKCB                 24    0.190  22.245   1.079   0.016   0.005   0.979
E K  4EKCB                 25    0.655 134.733   0.021   0.502   0.002   0.495
E Y  4EKCB                 26    0.458  97.810  -0.265   0.600   0.003   0.397
B G  4EKCB                 27    0.053   4.210   0.224   0.923   0.002   0.076
B L  4EKCB                 28    0.123  22.521   0.970   0.970   0.001   0.030
E A  4EKCB                 29    0.487  53.678   0.834   0.970   0.001   0.030
B A  4EKCB                 30    0.165  18.216   1.916   0.970   0.001   0.030
B F  4EKCB                 31    0.016   3.151   1.438   0.988   0.000   0.012
B R  4EKCB                 32    0.220  50.311   1.854   0.988   0.000   0.012
E A  4EKCB                 33    0.495  54.516   1.783   0.970   0.001   0.030
B F  4EKCB                 34    0.095  19.087   1.054   0.970   0.001   0.030
B L  4EKCB                 35    0.033   5.969   0.213   0.923   0.002   0.076
E K  4EKCB                 36    0.547 112.477   1.702   0.858   0.002   0.139
E S  4EKCB                 37    0.642  75.278   0.810   0.782   0.003   0.216
E E  4EKCB                 38    0.410  71.662   0.187   0.406   0.004   0.590
E F  4EKCB                 39    0.420  84.194  -0.959   0.176   0.004   0.820
B C  4EKCB                 40    0.159  22.352  -0.974   0.176   0.004   0.820
E E  4EKCB                 41    0.415  72.535  -1.126   0.406   0.004   0.590
E E  4EKCB                 42    0.336  58.734  -0.311   0.502   0.002   0.495
B N  4EKCB                 43    0.090  13.220   1.095   0.717   0.014   0.269
B I  4EKCB                 44    0.045   8.417   0.866   0.938   0.007   0.055
E E  4EKCB                 45    0.383  66.893   1.278   0.970   0.001   0.030
B F  4EKCB                 46    0.027   5.499   0.833   0.970   0.001   0.030
B W  4EKCB                 47    0.040   9.596   0.870   0.970   0.001   0.030
B L  4EKCB                 48    0.226  41.326   1.088   0.970   0.001   0.030
B A  4EKCB                 49    0.144  15.902   1.073   0.970   0.001   0.030
B C  4EKCB                 50    0.017   2.373   1.394   0.988   0.000   0.012
B E  4EKCB                 51    0.304  53.179   0.754   0.970   0.001   0.030
E D  4EKCB                 52    0.464  66.906   1.008   0.923   0.002   0.076
B F  4EKCB                 53    0.065  12.965   0.741   0.858   0.002   0.139
E K  4EKCB                 54    0.316  64.898   1.076   0.694   0.003   0.303
E K  4EKCB                 55    0.622 127.945   0.781   0.406   0.004   0.590
E T  4EKCB                 56    0.333  46.243   0.211   0.246   0.004   0.750
E K  4EKCB                 57    0.575 118.360  -0.648   0.109   0.005   0.886
E S  4EKCB                 58    0.571  66.956  -0.190   0.053   0.005   0.942
E P  4EKCB                 59    0.424  60.151   0.547   0.858   0.002   0.139
E Q  4EKCB                 60    0.699 124.752   0.209   0.923   0.002   0.076
E K  4EKCB                 61    0.491 100.978   1.507   0.923   0.002   0.076
B L  4EKCB                 62    0.066  12.158   0.602   0.970   0.001   0.030
E S  4EKCB                 63    0.291  34.140   1.401   0.970   0.001   0.030
E S  4EKCB                 64    0.570  66.804   1.059   0.988   0.000   0.012
B K  4EKCB                 65    0.236  48.504   1.549   0.988   0.000   0.012
B A  4EKCB                 66    0.016   1.719   1.013   0.988   0.000   0.012
E R  4EKCB                 67    0.407  93.272   2.093   0.988   0.000   0.012
E K  4EKCB                 68    0.436  89.603   2.180   0.988   0.000   0.012
B I  4EKCB                 69    0.038   6.975   0.407   0.988   0.000   0.012
B Y  4EKCB                 70    0.100  21.455   1.054   0.970   0.001   0.030
E T  4EKCB                 71    0.542  75.120   1.531   0.970   0.001   0.030
E D  4EKCB                 72    0.397  57.150   1.937   0.923   0.002   0.076
B F  4EKCB                 73    0.080  16.016   0.798   0.858   0.002   0.139
B I  4EKCB                 74    0.085  15.799   0.474   0.600   0.003   0.397
E E  4EKCB                 75    0.428  74.807   0.818   0.246   0.004   0.750
E K  4EKCB                 76    0.563 115.871  -0.157   0.053   0.005   0.942
E E  4EKCB                 77    0.760 132.842  -0.567   0.016   0.005   0.979
B A  4EKCB                 78    0.202  22.271  -1.518   0.018   0.019   0.964
E P  4EKCB                 79    0.581  82.444  -1.185   0.018   0.047   0.935
E K  4EKCB                 80    0.476  97.934   0.223   0.018   0.088   0.893
E E  4EKCB                 81    0.432  75.488  -0.273   0.018   0.088   0.893
E I  4EKCB                 82    0.470  86.950  -0.840   0.004   0.138   0.858
E N  4EKCB                 83    0.479  70.096  -0.539   0.005   0.045   0.951
B I  4EKCB                 84    0.125  23.107   0.036   0.018   0.019   0.964
E D  4EKCB                 85    0.525  75.710   0.762   0.176   0.004   0.820
E F  4EKCB                 86    0.590 118.393  -0.188   0.923   0.002   0.076
E Q  4EKCB                 87    0.525  93.676   1.135   0.970   0.001   0.030
B T  4EKCB                 88    0.053   7.323  -0.007   0.970   0.001   0.030
B K  4EKCB                 89    0.214  44.040   1.737   0.970   0.001   0.030
E T  4EKCB                 90    0.530  73.497   1.669   0.988   0.000   0.012
B L  4EKCB                 91    0.220  40.337   1.875   0.988   0.000   0.012
B I  4EKCB                 92    0.020   3.774   0.898   0.988   0.000   0.012
E A  4EKCB                 93    0.439  48.422   1.737   0.970   0.001   0.030
E Q  4EKCB                 94    0.658 117.572   1.286   0.923   0.002   0.076
B N  4EKCB                 95    0.209  30.642   0.291   0.782   0.003   0.216
B I  4EKCB                 96    0.116  21.497  -0.666   0.406   0.004   0.590
E Q  4EKCB                 97    0.721 128.717   0.604   0.176   0.004   0.820
E E  4EKCB                 98    0.668 116.682   0.442   0.109   0.005   0.886
B A  4EKCB                 99    0.183  20.222  -0.911   0.053   0.005   0.942
E T  4EKCB                100    0.272  37.713   0.544   0.016   0.005   0.979
E S  4EKCB                101    0.362  42.403   0.961   0.782   0.003   0.216
E G  4EKCB                102    0.523  41.168   1.040   0.879   0.010   0.111
B C  4EKCB                103    0.048   6.767   1.155   0.938   0.007   0.055
B F  4EKCB                104    0.031   6.262   0.524   0.970   0.001   0.030
E T  4EKCB                105    0.360  49.918   1.753   0.970   0.001   0.030
E T  4EKCB                106    0.373  51.707   2.010   0.988   0.000   0.012
B A  4EKCB                107    0.022   2.435   0.805   0.988   0.000   0.012
B Q  4EKCB                108    0.100  17.806   0.553   0.988   0.000   0.012
E K  4EKCB                109    0.492 101.184   1.905   0.988   0.000   0.012
E R  4EKCB                110    0.314  71.952   2.200   0.988   0.000   0.012
B V  4EKCB                111    0.013   1.967   1.688   0.988   0.000   0.012
B Y  4EKCB                112    0.185  39.428   1.284   0.988   0.000   0.012
E S  4EKCB                113    0.554  64.894   1.821   0.970   0.001   0.030
B L  4EKCB                114    0.192  35.210   2.046   0.970   0.001   0.030
B M  4EKCB                115    0.059  11.866   0.426   0.923   0.002   0.076
E E  4EKCB                116    0.487  85.114   0.631   0.782   0.003   0.216
E N  4EKCB                117    0.618  90.461   0.516   0.600   0.003   0.397
E N  4EKCB                118    0.485  70.960  -0.096   0.406   0.004   0.590
B S  4EKCB                119    0.221  25.866   0.098   0.321   0.003   0.675
B Y  4EKCB                120    0.126  26.905   0.654   0.246   0.004   0.750
E P  4EKCB                121    0.475  67.417   0.511   0.246   0.004   0.750
E R  4EKCB                122    0.458 104.928   1.131   0.321   0.003   0.675
B F  4EKCB                123    0.088  17.682  -0.658   0.339   0.016   0.645
B L  4EKCB                124    0.209  38.323   0.250   0.339   0.016   0.645
E E  4EKCB                125    0.522  91.176   0.293   0.257   0.016   0.727
B S  4EKCB                126    0.131  15.341  -0.192   0.522   0.016   0.462
E E  4EKCB                127    0.722 126.168   0.868   0.923   0.002   0.076
E F  4EKCB                128    0.252  50.496   1.315   0.970   0.001   0.030
B Y  4EKCB                129    0.048  10.364   0.440   0.970   0.001   0.030
E Q  4EKCB                130    0.327  58.331   2.152   0.970   0.001   0.030
E D  4EKCB                131    0.568  81.878   2.019   0.970   0.001   0.030
B L  4EKCB                132    0.087  15.875   1.086   0.970   0.001   0.030
B C  4EKCB                133    0.131  18.449   0.534   0.923   0.002   0.076
E K  4EKCB                134    0.649 133.396   1.485   0.858   0.002   0.139
E K  4EKCB                135    0.653 134.240   1.411   0.694   0.003   0.303
E P  4EKCB                136    0.443  62.848   0.113   0.502   0.002   0.495
E Q  4EKCB                137    0.884 157.865   1.028   0.016   0.005   0.979

Explain the output.  Go back.
```

---
